# Transient elastography correlated to four different histological fibrosis scores in children with liver disease

**DOI:** 10.1007/s00431-021-04001-6

**Published:** 2021-03-11

**Authors:** Ulrike Teufel-Schäfer, Christa Flechtenmacher, Alexander Fichtner, Georg Friedrich Hoffmann, Jens Peter Schenk, Guido Engelmann

**Affiliations:** 1 Department of General Pediatrics, Adolescent Medicine and Neonatology, Faculty of Medicine, Medical Center, University of Freiburg, Mathildenstr. 1, 79106 Freiburg, Germany; 2 Division of Pediatric Gastroenterology and Hepatology, Center for Child and Adolescent Medicine, University Hospital Heidelberg, Im Neuenheimer Feld 430, 69120 Heidelberg, Germany; 3 Department of Pathology, University Medical Centre, University of Heidelberg, Im Neuenheimer Feld 224, 69120 Heidelberg, Germany; 4 Division of Pediatric Radiology, Department of Diagnostic and Interventional Radiology, University Medical Centre, University of Heidelberg, Heidelberg, Germany; 5 Department of Pediatrics, Lukas Hospital, Neuss, Germany

**Keywords:** Pediatric, Transient elastography, Liver fibrosis, Histological scoring

## Abstract

Currently, liver histology is the gold standard for the detection of liver fibrosis. In recent years, new methods such as transient elastography (TE) have been introduced into clinical practice, which allow a non-invasive assessment of liver fibrosis. The aim of the present study was to investigate the predictive value of TE for higher grade fibrosis and whether there is any relevance which histologic score is used for matching. For this purpose, we compared TE with 4 different histologic scores in pediatric patients with hepatopathies. Furthermore, we also determined the aspartate aminotransferase-to-platelet ratio (APRI) score, another non-invasive method, to investigate whether it is equally informative. Therefore, liver fibrosis in 75 children was evaluated by liver biopsy, TE and laboratory values. Liver biopsies were evaluated using four common histological scoring systems (Desmet, Metavir, Ishak and Chevalier’s semi-quantitative scoring system). The median age of the patients was 12.3 years. TE showed a good correlation to the degree of fibrosis severity independent of the histological scoring system used. The accuracy of the TE to distinguish between no/minimal fibrosis and severe fibrosis/cirrhosis was good (*p* = 0.001, AUC-ROCs > 0.81). The optimal cut-off value for the prediction of severe fibrosis was 10.6 kPa. In contrast, the APRI score in our collective showed no correlation to fibrosis.

*Conclusion*: TE shows a good correlation to the histological findings in children with hepatopathy, independent of the used histological scoring system.

**Table:** 

**What is Known:** • *The current gold standard for detecting liver fibrosis is liver biopsy. Novel non-invasive ultrasound-based methods are introduced to clinical diagnostics.* • *Most histological scores have been developed and evaluated in adult populations and for only one specific liver disease.* **What is New:** • *Transient elastography (TE) in children showed a good correlation to fibrosis severity irrespective of the utilized histological scoring system.* • *The aspartate aminotransferase-to-platelet ratio (APRI) showed no correlation with different stages of liver fibrosis in children.*

**What is Known:**

• *The current gold standard for detecting liver fibrosis is liver biopsy. Novel non-invasive ultrasound-based methods are introduced to clinical diagnostics.*

• *Most histological scores have been developed and evaluated in adult populations and for only one specific liver disease.*

**What is New:**

• *Transient elastography (TE) in children showed a good correlation to fibrosis severity irrespective of the utilized histological scoring system.*

• *The aspartate aminotransferase-to-platelet ratio (APRI) showed no correlation with different stages of liver fibrosis in children.*

## Introduction

Liver fibrosis and liver cirrhosis are gradual changes in the liver structure caused by chronic liver disease [1]. These structural changes usually develop slowly over years, with liver cirrhosis being the final stage of fibrotic liver disease. The clinical progression from liver fibrosis to cirrhosis may be preventable if fibrosis is detected early enough.

The current gold standard for the detection of structural changes in the liver is histological evaluation. Several histological scoring systems have been established for the classification (necroinflammatory activity) and staging (fibrosis) of liver damage [2]. However, liver biopsy also carries potential risks, especially bleeding, infection, perforation of other organs and, especially in children, the risk of sedation [3]. Sampling errors, such as sample size or puncturing away from relevant areas of the liver, can also lead to an underestimation of liver damage [4, 5].

Therefore, a reliable non-invasive method for the detection of liver fibrosis and liver cirrhosis is needed in clinical practice. In addition to serological tests, e.g. aspartate aminotransferase-to-platelet ratio (APRI), ultrasound-based systems for the direct or indirect determination of liver stiffness have been introduced into clinical routine. Transient elastography (TE) is based on the measurement of the velocity of a shear wave induced by a mechanical impulse in the liver. The velocity of the shear wave reflects the stiffness of the liver. The stiffness depends primarily on the amount of extracellular matrix in the liver. Therefore, liver stiffness is strongly related to the extent of fibrosis. Reference values for TE in children were published earlier by our group [6].

The aim of the present study was to investigate the predictive value of TE for higher grade fibrosis and whether there is any relevance which histologic score is used for matching. For this purpose, we compared TE with 4 different histologic scores. Furthermore, we also determined the aspartate aminotransferase-to-platelet ratio (APRI) score, another non-invasive method, to investigate whether it is equally informative.

## Materials and methods

### Patients

This cohort study at a tertiary center included all patients who underwent liver biopsy in our department. The anthropometric data of the patients (age, sex, weight and height) were measured at the time of biopsy. The body mass index (BMI) and the BMI standard deviation score (BMI-SDS) were calculated. The study was conducted in accordance with the principles of the Declaration of Helsinki and approved by the local ethics committee (S-510/2009). Written informed consent was obtained from all patients.

### Evaluation of biomarkers of hepatic fibrosis and liver enzyme testing

In all patients, a fasting blood sample was taken on the day of the liver biopsy. Alanine aminotransferase (ALT), aspartate aminotransferase (AST), gamma-glutamyl transferase (GGT), cholinesterase (CHE), albumin, total and direct bilirubin, international normalized ratio (INR) and platelets were determined. APRI was calculated as AST/ULN × 100 divided by platelets (109/L) according to Wai et al. [7]. The upper limit of normal (ULN) was used according to the manufacturer’s recommendations. According to Wai et al., an APRI score of less than 0.5 means the absence of significant fibrosis in 85% of patients, while an APRI score above 1.5 is associated with significant fibrosis in 88% of patients.

### Ultrasound-based liver stiffness measurement: transient elastography

Liver stiffness was determined with the FibroScan® (Echosens, Paris, France). The measurement was performed in all patients within 3 months before or after the liver biopsy. The measurement was performed by placing the probe in the 7th or 8th intercostal space in the right ventral axillary line. The technique has been described in detail before [8]. Only measurements with ten valid shots, a success rate (ratio of the number of successful shots to the total number of shots) of at least 60% and an interquartile range < 0.3 were included. The median of all successful shots was automatically calculated by the device.

TE was performed throughout by the same experienced examiner (U.T.). Depending on the thoracic circumference, different probes (M-, S-probes) were used. As recommended by the manufacturer, the M-probe (3.5 mHz, diameter 7 mm) is used for children with a thoracic circumference of more than 75 cm. The S-probe (5 MHz, diameter 5 mm) has two different modes (S1, S2). The S1-mode allows the examiner to take measurements in infants with a thoracic circumference of less than 45 cm. The S2-mode is used for patients with a thoracic circumference of 45–75 cm. The measurement depth is 25–65 mm with the M-probe, 15–40 mm with S1-probe and 20–50 mm with the S2-probe.

### Liver histology

Liver biopsies were performed using the Menghini method [9] under deep sedation with propofol. Biopsies were performed by two experienced examiners (U.T., G.E.) with a 17-gauge needle. The specimen length was 2 cm or longer, with at least 10 portal fields aimed at. The liver tissue was fixed in formalin and embedded in paraffin. The samples were stained with hematoxylin and eosin and then evaluated by an experienced hepatopathologist (C.F.). A second experienced pathologist was consulted for a double evaluation.

The following four different scoring systems were used for staging liver fibrosis:

1. Desmet score [10]: Fibrosis is qualitatively assessed on a scale of 0–4. F0: no fibrosis, F1: portal fibrosis, F2: fibrosis with septa without disturbance of liver architecture, F3: septal fibrosis with severe disturbance of liver architecture, F4: cirrhosis.
2. METAVIR score [11] evaluates fibrosis qualitatively on a scale of 0–4. F0: absence of fibrosis, F1: portal fibrosis without septa, F2: portal fibrosis with few septa, F3: architectural distortion with numerous septa without cirrhosis, F4: cirrhosis.
3. Ishak score [12]: Fibrosis is qualitatively assessed on a scale of 0–6. F0: no fibrosis, F1: portal fibrosis of single portal fields, F2: portal fibrosis > 50% of portal fields, F3: plus single fibrotic portoportal bridges, F4: plus bridging fibrosis, F5: incomplete cirrhosis, F6: complete cirrhosis.
4. The semi-quantitative scoring system of Chevalier (SQS) [13] should be used independently of the etiology of fibrosis. It is mainly used in clinical studies and not in clinical practice. It is based on a scoring system that includes central vein, perisinusoidal fibrosis, portal field, number and size of septa.

All histological scores were further combined into a modified categorization system based on 3 levels: no/minimal fibrosis (NF), moderate fibrosis (MF) and severe fibrosis/cirrhosis (C), as shown in Table 1.

**Table 1:** Grading of fibrosis in no/minimal fibrosis, moderate fibrosis and severe fibrosis/cirrhosis to the histological scoring

	No/minimal fibrosis	Moderate fibrosis	Severe fibrosis/cirrhosis
Desmet	F0, F1 (*n* = 24; 32%)	F2 (*n* = 37; 49%)	F3, F4 (*n* = 14; 19%)
Metavir	F0, F1 (*n* = 25; 33%)	F2 (*n* = 35; 47%)	F3, F4 (*n* = 15; 20%)
Ishak	0, 1 (*n* = 19; 25%)	2–3 (*n* = 41; 55%)	4–6 (*n* = 15; 20%)
SQS	0–3 (*n* = 22; 29%)	4–11 (*n* = 42; 56%)	12–17 (*n* = 11; 15%)

*SQS* semi-quantitative scoring system of Chevalier

### Statistical analysis

IBM SPSS 20 for Windows (SPSS INC., Chicago, IL, USA) was used for statistical analyses. Unless stated otherwise, continuous variables are presented as median with range/interquartile range (IQR) or number (percentage). Differences between groups were tested by Student’s *t*-test or, if normality failed, with the Kruskal–Wallis (with Dunn’s post hoc test in case of multiple testing) or Mann–Whitney *U* rank-sum test. *p* values < 0.05 were regarded as statistically significant in a descriptive sense. The diagnostic performance of TE was assessed by receiver operating characteristics (ROC) curve analysis. Univariate and multivariate logistic regression analyses were performed to analyze predictors affecting TE. Variables were included in the multivariate model using a forward stepwise selection method, with a significance level of *p* < 0.1 for entering a variable into the model and *p* ≥ 0.2 for removal of a previously selected variable. To determine the optimal cut-off values that differentiate between NF and MF in combination with C, the Youden index method (maximum sensitivity and specificity) was used.

## Results

A total of 80 patients were enrolled in the study. The indication for liver biopsy was suspected liver disease. We excluded 5 patients with juvenile hemochromatosis, bile duct atresia or acute liver failure. The characteristics of the study population are shown in Table 2. The time interval between liver biopsy and TE ranged from the same day to 3 months (median 0 days).

**Table 2:** Characteristics of included patients at the time of liver biopsy

Characteristics	Patients (*n* = 75)
Gender (female), *n* (%)	33 (44%)
Age (years), mean (range) 0–11 years (prepubertal), *n* (%) 12–18 years, *n* (%) > 18 years, *n* (%)	12.3 (0.4–21.5) 37 (49.3) 32 (42.7) 6 (8.0)
BMI-SDS, mean (range)	− 0.01 (− 4.27–3.15)
Diagnoses, *n* (%)	
Autoimmune hepatitis	14 (18.7)
NASH	10 (13.3)
Wilson’s disease	8 (10.7)
Liver transplantation	8 (10.7)
Metabolic liver disease	9 (12)
Hepatopathy of unknown origin	19 (25.3)
Others^*^	7 (9.3)
ALT [U/l], mean (range)	271 (9–3884)
AST [U/l], mean (range)	217 (8–2706)

*BMI-SDS* BMI standard deviation score, *NASH* non-alcoholic steatohepatitis

*Autosomal recessive polycystic kidney disease (ARPKD), PSC, hepatopathy in chronic intestinal pseudoobstruction, hepatitis B and hepatopathy in Wegener’s granulomatosis

### Liver histology and performance of TE

According to the defined quality standards, TE was successfully performed on all patients. The procedure was fast (170 s, range 79–463 s), well tolerated and without complications. The median success rate was 100% (63–100%). Liver biopsies were performed in all children without complications. In all cases, an adequate sample could be taken.

In all groups, significant differences were found when comparing the liver stiffness of the TE results of the groups no fibrosis (NF) vs cirrhosis (C) (*p* = 0.001) and the groups moderate fibrosis (MF) vs C (*p* = 0.002–0.012). The results of TE and APRI stratification in NF, MF and C for each histological score are presented in Table 3. Between the four histological scoring systems investigated, there were no statistically significant NF-internal group differences in liver stiffness (*p* = 0.923), APRI (*p* = 0.976) and age (*p* = 0.953). Comparable results were found in the MF group (stiffness: *p* = 0.983, APRI: *p* = 0.802, age: *p* = 0.961) and in the C group (stiffness: *p* = 0.878, APRI: *p* = 0.992, age: *p* = 0.742).

**Table 3:** Results of TE in kilopascal expressed as median and IQR and APRI expressed as median and range for NF, MF and C of each histological score

	NF	MF	C	*p* value^#^	NF vs MF	NF vs C	MF vs C
					Post hoc*		
TE-D (kPa)	5.75 (4.8–8.9)	7.1 (5.8–14.5)	18.65 (10.4–36.3)	*p* < 0.001	*p* = 0.350	*p* = 0.0001	*p* = 0.012
TE-M (kPa)	6.1 (4.8–9.1)	6.8 (5.8–12.7)	20.0 (11.4–32.4)	*p* < 0.001	*p* = 0.862	*p* = 0.0001	*p* = 0.003
TE-I (kPa)	5.3 (4.6–8.1)	7.1 (5.8–13.2)	20.0 (11.4–32.4)	*p* < 0.001	*p* = 0.153	*p* = 0.0001	*p* = 0.004
TE-S (kPa)	5.75 (4.8–8.8)	6.95 (5.8–15.3)	24.9 (11.4–48.0)	*p* < 0.001	*p* = 0.293	*p* = 0.0001	*p* = 0.002
APRI-D	0.54 (0.19–0.99)	0.73 (0.45–1.44)	0.78 (0.4–3.01)	*p* = 0.200	Not applicable		
APRI-M	0.57 (0.19–1.25)	0.65 (0.44–1.17)	0.8 (0.42–3.08)	*p* = 0.271	Not applicable		
APRI-I	0.44 (0.19–1.05)	0.65 (0.45–1.17)	0.8 (0.42–3.08)	*p* = 0.161	Not applicable		
APRI-S	0.47 (0.18–0.87)	0.73 (0.45–1.77)	0.8 (0.42–3.08)	*p* = 0.097	Not applicable		

*APRI* aspartate aminotransferase-to-platelet ratio, *C* severe fibrosis/cirrhosis, *D* Desmet, *I* Ishak, *M* Metavir, *MF* moderate fibrosis, *n.s.* no significance, *NF* no/minimal fibrosis, *p* = *p* value, *kPa* kilopascal, *S* semi-quantitative scoring system of Chevalier, *TE* transient elastography

^#^Kruskal–Wallis test

*Dunn’s post hoc test

### Predictive performance of TE (ROC curve analysis)

**Fig. 1:**
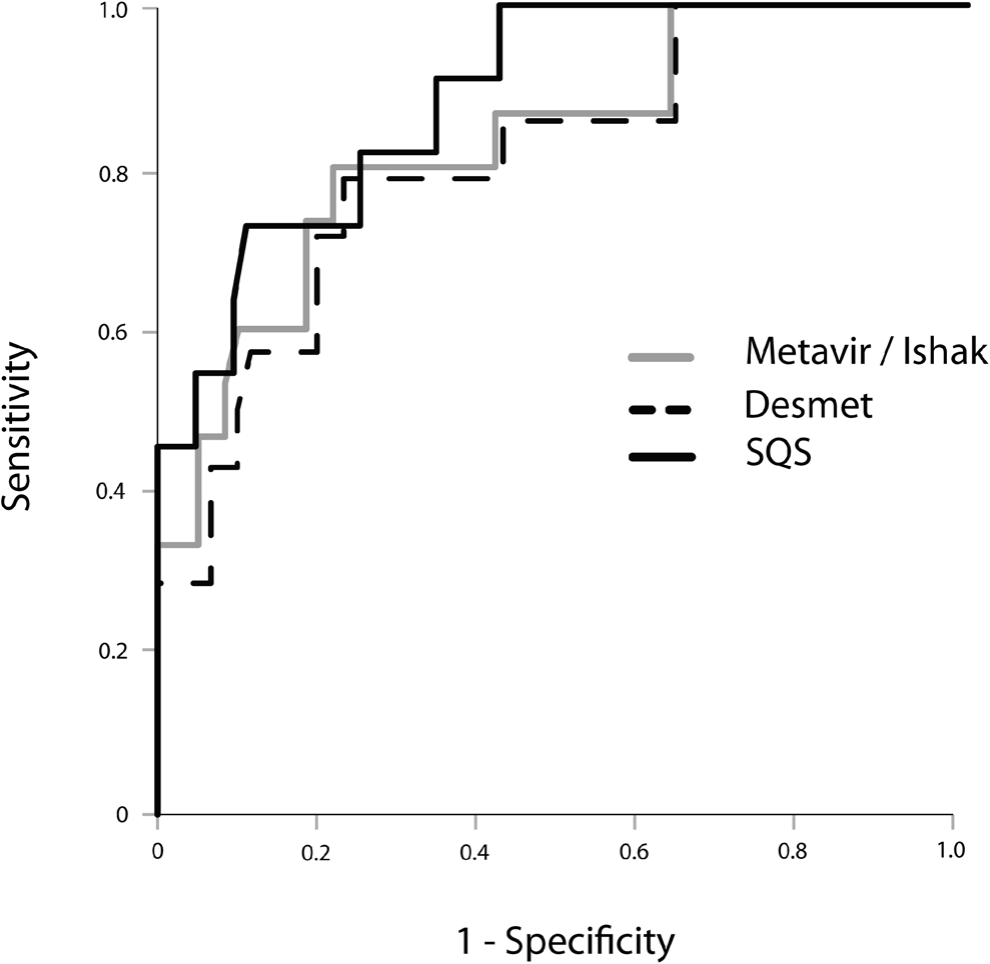
ROC curve for discrimination between no/moderate fibrosis and severe fibrosis/cirrhosis by TE stratified according to different fibrosis scoring systems. Desmet-AUC = 0.81 [0.68–0.94]; Metavir/Ishak-AUC = 0.83 [0.71–0.90]; SQS-AUC = 0.89 [0.80–0.98]. AUC area under the curve, SQS semi-quantitative scoring system of Chevalier

The accuracy of TE for the detection of MF and C was good in all histological scoring groups (AUC 0.81–0.89). Details on AUC-ROCs and 95% confidence intervals for the different histological grading scores are given in Fig. 1. Liver stiffness cut-off values (Youden index) were 10.6 kPA for Desmet, Metavir and Ishak and 17.6 kPA for SQS.

### Factors associated with an increased TE (regression analysis)

In addition, we performed univariate linear regression to determine the relationship between TE and clinical indices of patients, including age, BMI-SDS, ALT, total bilirubin, albumin, CHE, INR and platelet. There was a positive correlation between total bilirubin, INR and TE. Serum albumin, CHE and platelets were negatively correlated with TE. To determine factors that are independently associated with liver fibrosis, a multivariable linear regression analysis was performed, which showed that CHE, total bilirubin and platelets were significantly associated with higher TE values (Table 4).

**Table 4:** Linear regression model to identify factors associated with increased liver stiffness

	Univariate		Multivariate	
	Coefficient	*p* value	Coefficient	*p* value
Age	0.091	0.540		
BMS-SDS	− 0.427	0.471		
ALT	0.001	0.419		
Bilirubin	0.709	< 0.001	0.571	0.015
Albumin	− 0.365	0.036		
CHE	− 1.792	< 0.001	− 1.040	0.033
INR	23.624	0.001		
Platelet	− 0.021	< 0.001	− 0.021	0.022

*BMS-SDS* body mass index standard deviation score, *ALT* alanine aminotransferase, *CHE* cholinesterase, *INR* international normalized ratio

## Discussion

The prognosis of chronic liver disease depends on the development of fibrosis and/or cirrhosis [14]. The gold standard for the definition of fibrosis stages is histology. However, most histological scores have only been developed for specific liver diseases in adult patients. For example, the Metavir score [11] has been validated in patients with hepatitis C. It is based on a non-quantitative analysis of portal and periportal fibrosis. It is also used for hepatitis B and autoimmune hepatitis, as these diseases have a similar distribution of fibrosis. In other liver diseases, fibrosis is more perivenular or perisinusoidal. Therefore, the adequacy of the Metavir score in these diseases must be questioned. The situation is similar to the Desmet and Ishak scores. In contrast, the semi-quantitative score developed by Chevallier et al. [13] includes the extent of fibrous deposits in all compartments of the liver. It can therefore be applied to any type of chronic liver disease. However, it is rarely used in clinical routine. Although the above-mentioned scores have only been developed and evaluated in adults, they are also used in children.

Several studies have compared TE and liver biopsy in adult populations [8, 15–20]. For example, in pediatric studies, the Metavir score has been used to assess TE in children with biliary atresia, intestinal failure, total parenteral nutrition and in children after liver transplantation [21–24]. The Ishak score has been used in pediatric populations following liver transplantation [24, 25] and in viral hepatitis [26]. To our knowledge, there is only one study besides Metavir in which SQS is used to evaluate elastography [27]. In this study, 33 children underwent liver biopsy. The SQS varied between 1 and 23 (median: 10), with 11 patients having a score above 15. TE values correlated significantly with Metavir fibrosis stages (Kendall coefficient 0.53; *p* < 0.001) and SQS (Kendall coefficient 0.49; *p* < 0.001). To date, no pediatric study has been published that evaluates the TE value and uses the Desmet score.

Furthermore, there is no consensus on a histological scoring system for the classification of childhood liver fibrosis that is adapted to the different types of liver diseases in children. Therefore, we investigated TE in correlation with four widely used histological scores including a semi-quantitative test. Our results show that TE has a good correlation to each of the four systems. The accuracy of TE for differentiation between NF and C was good, with AUC-ROCs of more than 0.81 in severe fibrosis/cirrhosis.

The median liver stiffness increased gradually depending on the degree of fibrosis. It had no influence on which histological score was used. However, the TE could not distinguish between different low degrees of fibrosis. Of course, it would be advantageous if even minor stages of fibrosis could be distinguished by TE. Thus, depending on the underlying disease, further progression of fibrosis to cirrhosis could be avoided by adjusting the therapy.

With regard to the differentiation of the different stages of fibrosis, the results of the published data are contradictory: Behairy et al. [26], who used the Ishak score, reported a good, significant differentiation of earlier fibrosis stages, while Friedrich-Rust et al. [28] and de Ledinghen et al. [27], who used the Metavir score, only reported a good performance in differentiating between cirrhosis and low-grade fibrosis, but not between different low grades of fibrosis. One reason could be the wider range of the Ishak score (7 stages) compared to the Metavir score (5 stages) or our aggregation (3 stages).

The optimal cut-off value for the diagnosis of cirrhosis was 10.6 kPa for Metavir, Desmet and Ishak and 17.6 kPa for SQS. This is comparable with data from adult studies [29]. So far, there are few studies that deal with cut-off values in childhood. Ledighen et al. [27] describe in their study a comparable cut-off of 9.9 +/− 0.6 kPa for various liver diseases. However, only 33 (28%) of 116 children received a liver biopsy. In his cohort, the distribution in the fibrosis groups was very different. None had F0, and 54% had an F3–F4 Metavir score. In an Italian study with 52 children with advanced fibrosis, a cut-off of 10.2 kPA was found [30]. Compared to our study, Nobili included children from the age of 4 years, the cohort size was smaller and all children had non-alcoholic steatohepatitis. There is a study by Lee et al. [31] with a similarly heterogeneous study group as ours and comparable cohort size. However, patients with cholestasis were not excluded here. Lee et al. [31] give an optimal cut-off of 8.6 kPa to distinguish advanced fibrosis from low fibrosis. The lower value could be due to the fact that 25% of the TE measurements in this group were performed more than 6 months after the liver biopsy. In our cohort, the time interval between liver biopsy and TE ranged from the same day to 3 months (median 0 days).

Good accuracy of simple non-invasive tests such as the APRI score for the prediction of liver fibrosis and cirrhosis in various primary liver diseases has been demonstrated in several studies with adults [7, 32, 33]. In this study, we could not detect significant differences of the APRI between the histologically defined severity grades (fibrosis, cirrhosis) (Table 3). Hence, the predictive performance was low. There are controversial results in pediatric studies. De Ledinghen [27] and Kim [34] showed a significant correlation of the APRI score with histology in children with chronic liver disease, while Hukkinen [35] and Diaz [22] described the detection of cirrhosis by APRI but could not show a difference between the low stages of fibrosis. Interestingly, de Ledinghen [27] found a good correlation between APRI and Metavir stages, but not between APRI and SQS scores. Lind et al. [36] examined 31 children with biliary atresia. The APRI scores showed no difference between the Metavir categories. Our results are quite similar. The APRI score in our setting of different hepatopathies did not distinguish cirrhosis from grade 0/1 fibrosis. We could show a slight increase in APRI score with increasing fibrosis, but no significant correlation between APRI and the severity of fibrosis. There may be several reasons for this phenomenon. Changes in platelet count and AST develop late in the course of the disease, and AST may even be normal in patients with compensated cirrhosis. Another reason could be the upper limit of normality of AST, which is age-related and depends on the manufacturer’s recommendation [37]. To date, there are no international manufacturer-independent AST values.

To determine factors that are independently associated with liver fibrosis, we performed multivariate linear regression analysis. As a result, CHE, total bilirubin and platelets were independent variables associated with higher TE. Shan et al. [38] also described platelet count as a relevant factor. In their study, 181 adults with various liver diseases were included. Other relevant determinants were albumin, BMI and prothrombin activity. The liver enzymes had no correlation with fibrosis. However, these data are inconsistent. Tapper et al. [39] showed a correlation between increased ALT values and liver stiffness. However, these statistical phenomena do not help in the diagnosis of severe fibrosis or cirrhosis.

The present study has some limitations. One of the main limitations is the sample size with relatively small numbers, especially in the group with severe fibrosis/cirrhosis. In addition, our study cohort includes a heterogeneous group of liver diseases, although this group of diseases is quite representative for the hepatopathies in children that are also found in clinical practice.

TE is not proposed as an alternative to biopsy for diagnostic purposes. However, TE can be a useful tool for quickly and easily gaining an initial impression of structural changes in the liver. It also allows easy follow-up of already known structural changes in the liver. Therefore, a validated non-invasive marker for fibrosis could help to decide which patients need a biopsy to diagnose the underlying liver disease and could reduce the number of liver biopsies in children with chronic liver disease. In this study, we could show that there is a good correlation between staging of fibrosis and the results of TE regardless of the histological scoring system used. However, a significant distinction could not be shown at lower fibrosis stages. We could not demonstrate any significant differences of the APRI between the histologically defined severity grades in our very heterogeneous pediatric study population.

## Data Availability

Can be provided upon request.

## Abbreviations

ALT: Alanine aminotransferase
APRI: Aspartate aminotransferase-to-platelet ratio
ARPKD: Autosomal recessive polycystic kidney disease
AST: Aspartate aminotransferase
BMI: Body mass index
BMI-SDS: Body mass index standard deviation score
CHE: Cholinesterase
GGT: Gamma-glutamyl transferase
INR: International normalized ratio
IQR: Interquartile range
PSC: Primary sclerosing cholangitis
ROC: Receiver operating characteristics
SD: Standard deviation
SQS: Semi-quantitative scoring system of Chevalier
TE: Transient elastography
ULN: Upper limit of normal
